# Analysis of medical expenditure and socio-economic status in patients with ocular chemical burns in East China: a retrospective study

**DOI:** 10.1186/1471-2458-12-409

**Published:** 2012-06-06

**Authors:** Qihua Le, Yan Chen, Xin Wang, Jiaxu Hong, Xinghuai Sun, Jianjiang Xu

**Affiliations:** 1 Department of Ophthalmology, Eye & ENT Hospital of Fudan University, No. 83 Fenyang Road, Shanghai, 200031, China

**Keywords:** Burns, Chemical, Medical expenditure, Socio-economic status, China

## Abstract

****Background**:**

Little has been known regarding the relationship between ocular chemical injury and victims’ medical expenditure, income loss and socio-economic status changes. So we conduct this retrospective cross-sectional study in patients with ocular chemical burns in East China.

****Methods**:**

Fifty-six patients were enrolled and required to complete a self-report questionnaire consisting of the following contents: entire expenditure on medical treatment; the victims’ personal and household per capita income, and income loss caused by the injury; and the changes of socioeconomic status as well.

****Results**:**

The median expense of medical treatment was CNY 40,000 (approximately US$5,900). The medical expenditure rose significantly with increased injury severity, prolonged hospital stay, and increased frequency of surgery. More than half victims (51.8 %, 29/56) paid all or the majority of medical expense by themselves. The expense of only 5 victims was mainly paid by medical insurance, accounting for less than ten percent (8.9 %, 5/56). The victims’ personal and household per capita income both decreased significantly after the injury, with the median reduction being CNY 24,000 and CNY 7,800 (approximately US$3600 and US$1200) per year respectively. The reduction amplitude of personal and household per capita income rose with increased injury severity and prolonged time of care required. The injury caused emotional depression or anxiety in 76.8 % (43/56) victims, and the relationship with their relatives got worse in 51.9 % (29/56) patients. Moreover, only 21.4 % (12/56) patients felt that the whole society gave them care and concern after the injury, whereas 46.4 % (26/56) and 28.6 % (16/56) felt indifference or discrimination from society as a whole (*X*^2^ = 16.916, P = 0.028).

****Conclusions**:**

The medical expense was a huge economic burden to most victims of ocular chemical burns, and personal and household per capita income of the victims decreased significantly after injury, both of which had a close relationship with the injury severity. Formal legislation was urgently needed to compel the employer to purchase injury or medical insurance and provide more compulsory protection to the population working in high risk occupations. In addition, psychological counseling and instruction shouldn’t be neglected in the aid and treatment of victims.

## **Background**

Although chemical burn is a less common ocular injury, with a reported incidence ranging from 1.25‰ to 4.4‰ in developing countries [1-5], it usually causes devastating damage to the vision and accounts for a small but significant part of corneal blindness in China [6]. Severe chemical burn leads to complete destruction of the ocular surface, cornea opacification, permanent vision loss, and rarely loss of the eye [7]. Moreover, most victims are young men who earn the majority income for the family. The injuries not only cause loss or decrease of vision, either monocular or binocular, but also lead to difficulty in work, study, and daily life.

Chemical burns predominantly occurred at the workplace in China [4,5]. Although the Chinese government issued the regulation in 2004 that employers should provide injury insurance for their employees, its execution varied in different enterprises and areas. In the majority of state-owned enterprises, universities, institutions, or the enterprises in relatively developed areas, basic insurance was provided to the employee, covering medical and injury insurance. However, the enforcement of the regulation was fairly poor in many private enterprises or in underdeveloped areas due to the lack of supervision from local government. Moreover, healthcare insurance are not compulsory in China, and poorly-educated employees haven’t consciousness to buy healthcare insurance for themselves. Therefore, once chemical injury occurred the victims couldn’t get medical cost and compensation from insurance.

Although researchers have investigated the epidemiological and demographic issues of ocular chemical burns in different population groups including Chinese [1-5], little is known about the relationship between the chemical injury and expenditure on medical treatment, and the changes on the patients’ income and their socio-economic status after the injury. However, we consider that the study in this aspect has crucial public health implications and is important for proposing socio-economic strategies and administering economic and psychological assistance to minimize its impact on the working population at high risks. In view of this, we performed this retrospective study to investigate the expense on medical treatment in patients with chemical burns and to examine the changes in personal and household income and their socio-economic status by the application of a self-report questionnaire.

## **Methods**

This retrospective cross-sectional study enrolled 56 consecutive patients with ocular chemical burn, who were scheduled for vision care at the Department of Ophthalmology, Shanghai Eye, Ear, Nose, and Throat Hospital of Fudan University, the major eye center in East China, from January 1 through April 30, 2010. Eligibility criteria included having at least three months of clinical course to achieve relatively stable results of the visual outcome, having no other systemic or ocular disease that could potentially affect working capacity and daily activity, and being aged 16 years or older. The study was approved by the ethics committee of Shanghai Eye, Ear, Nose, and Throat Hospital and conducted according to the tenets of the Declaration of Helsinki. Informed consent was obtained from all patients.

Before answering the questionnaire, all patients underwent a routine ophthalmologic examination, including best-corrected visual acuity, slitlamp biomicroscopy, tonometry, and indirect ophthalmoscopy when feasible. The degree of the injury was assessed by an experienced cornea specialist under slitlamp biomicroscopy, according to the Hughes–Roper–Hall classification scale [8].

The questionnaire contains four parts with a total of eighteen questions, as listed in Table 1. The patients were asked to complete the questionnaire. Research staff explained the questionnaire to them, and provided assistance when required (for the participants whose eyesight was too poor to enable reading, research staff read the questionnaire for them, in a neutral and uniform manner, and recorded the patients’ choices). The completed questionnaires were reviewed by research staff to ensure no data were missing.

**Table 1:** The detailed content of the questionnaire used in the current study

**Category**	**Questions**	**Choice and appendix question**
Data on demography and injury	Q1.name, gender,occupation, education	
	Q2.injury agent	
	Q3.where injury occurred	
	Q4.monocular or binocular injury	
	Q5.hospitalization or not	If yes, the accumulative time of hospitalization
	Q6.operation or not	If yes, monocular or binocular? Single or multiple operations?
Expense on treatment	Q7.Total expense on medical treatment	
	Q8.The proportions of the expense on drug, examination, operation and hospitalization, and the nutrition and transportation respectively	
	Q9.The proportions of expense paid by the employer, insurance and patient himself respectively	
Loss of income	Q10.The personal income per year of the patient before and after the injury	If reduced, the main reason?
	Q11.The per capita income of the family per year before and after the injury	
	Q12.the time required for care from family members (hours per day)	
The impact of the injury on the socio-economic status of the patients	Q13.Working or studying capacity	A:without difficulty, B:doing simple work or study, C: losing working or studying ability (being umployed or dropping out of school)
	Q14.The ability to perform daily activities	A: without difficulty, B: being able to do simple activity such as eating and clothing, C: entirely losing ability to perform daily acitvity and need care and help from others
	Q15.Emotional anxiety or depression	A:without anxiety or depression, B: anxious or depressive somewhat, C: extremely anxious or depressive
	Q16.The negative impact on the education of victims' children (if they had)	A: without negative impact, B: to some extent, C: very serious
	Q17.The impact on the relationship between family members	A: no change,B: indifference, C: be discriminated, D: be discarded
	Q18.The attitude of whole society towards them after injury	A: giving care and concern, B: indifferent, C: generally be discriminated, D: other

After the adjustment of age and gender, either Mann–Whitney U test or a nonparametric analysis of variance (ANOVA) was performed to determine the differences on the medical expense paid by different subgroups, which were divided according to the severity and operation status. Comparisons on the personal and household per capita income of different subgroups were made by pair-wise *t*-test or Chi-square test. Spearman's correlation analysis was used to evaluate the correlation of patients’ expense and time of hospitalization, as well as time required for care from family members of patients and household income per year after injury. To examine the impact of the injury on the socio-economic status of the patients with different injury severity, a Chi-square test was used. All tests were considered statistically significant at P <0.05 (SPSS for Windows, version 13.0; SPSS, Inc., Chicago, IL).

## **Results**

A total of 91 eyes of 56 patients were included in the study, including 35 patients suffering bilateral injuries. The demographics of these subjects are shown in Table 2. The mean age of enrolled subjects was 37.84 ±11.67 years, with the majority being male (98.2 %) and factory workers (62.5 %). 30 patients were alkali burns, 20 were acid burns, and the others had been burnt by other chemical agents. Among all enrolled patients, 35 subjects underwent binocular chemical burn, the rest were monocularly injured.

**Table 2:** Demographic features of enrolled subjects

**feature**		**patients**
sex	male	55
	female	1
age(yrs)		37.84 ±11.67
		range 16-63
occupation	factory worker	35
	construction worker	11
	peasant	5
	student	3
	other	2
education	illiteracy	2
	primary school	13
	middle school	36
	university	4
	post-graduate	1
location where injury occurred	workplace	45
	laboratory	4
	home	5
	elsewhere	2

The median expense of medical treatment was CNY 40,000 (approximately US$5,900), ranging from CNY 4,000 (approximately US$590) to CNY 450,000 (approximately US$66,400). The average proportions of the expenditure on drugs, examination, operation and hospitalization, and nutrition and transportation were 19.8 %, 12.7 %, 53.3 %, and 14.2 %, respectively. Although the average medical expense for binocularly injured patients was significantly higher than for monocularly injured ones (Z = 2.653, P = 0.008), the proportion of expense for each part didn’t have statistical significance between the two groups, with the operation and hospitalization accounting for half of the total expense, as showed in Table 3. From the 56 enrolled subjects, 53 were treated in the inpatient department, with a median time of 22 days. Spearman's correlation analysis showed that the expenses paid by the patients were significantly correlated with the length of hospitalization (ρ = 0.430, P = 0.002).

**Table 3:** Total expense paid by the victims and the proportion of each part

	total expense (thousand CNY)*	**The proportion of each part in total expense (%)**			
		**drug**	**examination**	**operation and hospitalization**	**the nutrition and transportation**
total (n = 56)	40 (4–450)	19.8	12.7	53.3	14.2
monocular injury (n = 21)	25(4–60)	22.4	14.7	51.6	11.3
binocular injury (n = 35)	55(7.5-450)	18.8	12.1	53.8	15.3

* The average expense was expressed as median, and the range of expense was listed in the brackets.

Table 4 showed the expense analysis among patients with different clinical severity, revealing that bilaterally injured ones with the less severely burnt eye classified as Grade III or IV spent much more money on medical treatment than the other less severely injured ones (F = 3.393, P = 0.025). The median expense paid by unoperated, unilaterally operated, and bilaterally operated patients was CNY 20,000, 25,000 and 100,000 respectively (approximately US$3,000, 3,800 and 15,000). The analysis reveals that the expenses paid by bilaterally operated ones was significantly higher than those of others (F = 6.259 and 7.928, P = 0.003 and 0.002, respectively). Moreover, the median expense for patients undergoing single surgery and multiple surgeries was CNY 25,000 and 80,000 (approximately US$3,800 and 12,000), respectively, with the latter being significantly higher than the former.

**Table 4:** The average expense of patients with different clinical severity

**Group**	**total expense (thousand CNY)**	
group A (n = 9)	24.6 ± 16.5 (20)	
group B (n = 12)	41.9 ± 29.5 (30)	
group C (n = 16)	49.9 ± 48.3 (35)	
group D (n = 19)	93.8 ± 91.0 (75) *	compared with group A

The expense was expressed as average ± SD, with the median listed in the brackets.

* P < 0.05.

Group A: unilateral eye injured with the classfication as grade I or II.

Group B :unilateral eye injured with the classfication as grade III or IV.

Group C: bilateral eyes involved with the less severely injured eye classified as grade I or II.

Group D: bilateral eyes involved with the less severely injured eye classified as grade III or IV.

It’s notable that more than half the victims (51.8 %, 29/56) paid all medical expenses or the majority of expenses by themselves, including 21 workers, 5 farmers, 2 students, and one patient with another occupation. Moreover, the number of patients whose medical expense was mainly paid by medical insurance was only five, accounting for less than ten percent (8.9 %, 5/56). The medical expense of the other 22 patients, of whom 21 were workers, was paid entirely by their employers.

After the injury, the personal income of 39 victims (69.6 %) decreased, among whom 15 subjects lost all income. Similarly, the household income per capita decreased in 40 victims’ families (71.4 %), among which 7 didn’t have any income after the injury. The median reduction of personal income and household income per capita was CNY 24,000 and 7,800 (approximately US$3,600 and 1,200) per year, respectively. The differences in the income between pre- and post-injury both had statistical significance (t = 7.133 and 5.298, P = 0.002 and 0.007, respectively). It’s notable that in those with decreased personal and household income, the proportion of bilaterally injured victims was significantly higher than that of unilaterally injured ones, especially those with the less severely burned eye classified as Grade III or IV (*X*^2^ = 15.501 and 13.896, P = 0.008 and 0.017). Further analysis showed that the major reason for reduced income was unemployment due to the partial or entire loss of working ability caused by the injury, accounting for 59 % (23/39) of cases. It should be highlighted that in patients entirely losing the ability to perform daily activity, such as clothing and eating, both personal and household income decreased significantly (*X*^2^ = 14.131 and 18.503, P = 0.007 and 0.001). Moreover, the time of care from family members required for the disabled patients had a significant correlation with the reduction of household income (ρ = 0.637, P = 0.001).

The analysis of the socio-economic status changes of the patients after injury showed that 76.8 % (43/56) of victims became depressed or anxious, and the relationship with their relatives got worse for 51.9 % (29/56) of patients. What’s more, the emotional condition of the victims and their family relationship deteriorated with the increased severity of injury and the number of injured eyes (*X*^2^ = 21.825 and 20.402, P = 0.001 and 0.002). Only 21.4 % (12/56) of patients felt that the whole society offered care and concern after the injury, whereas 46.4 % (26/56) and 28.6 % (16/56) felt indifference or discrimination from the whole society (*X*^2^ = 16.916, P = 0.028). Although 53.6 % (30/56) patients reported that the injury produced a negative impact on their children’s education, statistically significant difference wasn’t found by the analysis (*X*^2^ = 8.219, P = 0.222), as shown in Table 5.

**Table 5:** The number of patients with different clinical severity making choices on the changes in socio-economic status after injury

		**Group A**	**Group B**	**Group C**	**Group D**	**Chi**	**P**
Emotional depression or anxiety	Choice A	6	2	4	1	21.825	0.001*
	Choice B	3	7	8	9		
	Choice C	0	3	2	11		
The negative impact on children's education	Choice A	4	8	7	7	8.219	0.222
	Choice B	5	3	5	8		
	Choice C	0	1	2	6		
The impact on family relationship	Choice A	8	10	4	5	20.402	0.002*
	Choice B	1	2	6	8		
	Choice C	0	0	4	8		
The social attitude towards them after injury	Choice A	2	4	5	1	16.916	0.028*
	Choice B	3	4	4	15		
	Choice C	3	4	4	5		
	Choice D	1	0	1	0		

* P < 0.05.

Group A: unilateral eye injured with the classification as grade I or II.

Group B :unilateral eye injured with the classification as grade III or IV.

Group C: bilateral eyes involved with the less severely injured eye classified as grade I or II.

Group D: bilateral eyes involved with the less severely injured eye classified as grade III or IV.

The content of each choice was listed in Table 1, from Q 15 to Q 18.

## **Discussion**

Chemical injury is not rare in China, especially in some occupations with high risk, such as factory and construction work. Most victims in the present study were young men, which was in agreement with previous studies [2-5]. The current study showed that the median expense of medical treatment paid by the chemical burn victims in Eastern China was approximately US$5,900, far exceeding the per capita GDP of China reported by the International Monetary Fund in 2010 (http://www.imf.org), which was 3566 US$. Moreover, according to the data revealed by the National Bureau of Statistics of China, disposable income per capita for urban and rural households in eastern China in 2010 were approximately US$2,900 and US$900, respectively (http://www.stats.gov.cn/tjgb/). The huge gaps between the income and expenditure would undoubtedly cause enormous economic burden to the victims and their family, if they hadn’t insurance and the expenses were mainly or entirely paid by themselves.

The current study showed that the medical expense rose with the increased injury severity, half of which paid on the surgery and hospitalization. The severity of ocular injury after a chemical exposure has a close relationship with the contact surface area and degree of penetration [9]. Rarely could mild chemical burn cause incurable visual impairment, and a large proportion of mildly injured victims didn’t require operation. In contrast, moderate or severe burns commonly lead to entire damage of ocular surface tissue, requiring long-term medical treatment and multiple surgeries [10-14]. In addition, the vision recovery process after multiple complicated surgeries was time-consuming, meaning for a longer hospital stay. Therefore, more severely injured patients were reasonably expected to pay more money on medical treatment, and their medical expense significantly correlated with the length of hospitalization.

Chemical burns predominantly occurred at the workplace in China [4,5,15], especially in privately owned enterprises where the protective devices were inadequate. The current study confirmed that few employees working in the private enterprises had their medical expense paid by the insurance. Moreover, once the injury occurred, most employers only paid the expense for the initial stage of the medical treatment and fired the victims after giving them a small or large amount of money, depending on the injury severity, as the compensation. However, the restoration of visual function is a time- and money-consuming process. Compensation from the employers could rarely cover the expense for following treatment without the support of medical insurance. Consequently, the victims had to pay for the majority of expenses by themselves, especially those severely injured, leading to a heavy economic burden to them and their family, and even causing them to abandon following treatment.

Another important impact of injury on the victims was that personal and household income decreased significantly after injury. Most of victims were unemployed or obliged to change their job after the injury due to visual impairment and consequent loss of working ability, being incapable of completing the work they used to do and causing reduction or even entire loss of personal income. Furthermore, income reduction had been shown to have a significantly close relationship with the injury severity and the time of care and help from relatives. The most severely injured ones not only completely lost working ability, but also had great difficulty in performing many daily activities, such as eating and clothing, and required all-day care and help from others, such as their relatives. Therefore, in order to look after the injured victim, at least one adult family member couldn’t go to work and the household income further decreased. The reduction of household income led to difficulty in spending more money on the medical treatment, forming a vicious cycle detrimental to the vision recovery of patients.

Our previous study demonstrated that most victims of chemical burns lacked education on the awareness of the dangerous properties of chemical agents and the correct methods of managing first aid measures after the injury [5,16]. Moreover, none of the victims wore eye-protection devices as a routine procedure, even though they believed that such protection was required and helpful. It should be highlighted that the average price of eye-protection glasses available in Chinese market is merely CNY 20 (US$ 3.0), forming a huge contrast compared with the cost of treatment and lost income. Given the cost effectiveness of eye-protection devices, formal education, reinforcement, compulsory use, and formal legislation should be enforced to improve compliance in using protective devices. Meanwhile, safer working practices, hazard warnings, and safety advice on chemical product packaging are also crucial for reducing the incidence of ocular chemical injury.

The present study revealed that the injury caused huge impact on the emotional health of the victims. The majority of them became depressed or anxious to some extent after injury, and had a feeling of indifference or discrimination from either their family members or the whole society. Not only visual disability, but also significant facial disfigurement, which usually accompanied with ocular chemical injury, contributed to negative emotional changes. Furthermore, the emotional condition deteriorated with the increased injury severity. Our findings indicated that, apart from medical treatment, the psychological counseling and instruction should be emphasized urgently in the aid and treatment of the victims, especially those severely injured.

There are several limitations in this study. One is that the times of surgeries and operation method in patients with multiple surgeries wasn’t taken into consideration in the analysis of medical expense. The main reason was that some patients lost their records of case history and couldn’t tell us the exact method of surgery. The other is limited number of enrolled subjects, hampering further regression analysis on the correlation between either medical expense or the income after injury and likely-related factors, such as chemical agent, educational level, occupation and injury severity. This shortcoming might be resolved in further investigation with a larger number of enrolled subjects.

## **Conclusions**

In summary, the medical expense was a huge economic burden to most victims of chemical burns in East China, and personal and household per capita income of the victims decreased significantly after injury, both of which had a close relationship with the injury severity. Formal legislation is urgently needed to compel employers to purchase injury or medical insurance and provide more protective devices to the population working in high risk occupations. In addition, the psychological counseling and instruction shouldn’t be neglected in the aid and treatment of the victim, and the whole society should be expected to give them more care, help, and support.

## **Competing interests**

'The authors declare that they have no competing interests.

## **Authors’ contributions**

Le Q collected the data and drafted the manuscript. Chen Y and Wang X collected the data. Hong J performed the statistical analysis. Sun X participated in the design of the study and gave linguistic help in manuscript preparation. Xu J played a predominant role in the design and conceptualization of the article. All authors have revised the manuscript critically for important intellectual content and have approved the final manuscript.

## Pre-publication history

The pre-publication history for this paper can be accessed here:

http://www.biomedcentral.com/1471-2458/12/409/prepub
